# Genomic epidemiology and characterization of *Staphylococcus aureus* isolates from raw milk in Jiangsu, China: emerging broader host tropism strain clones ST59 and ST398

**DOI:** 10.3389/fmicb.2023.1266715

**Published:** 2023-09-22

**Authors:** Hui Liu, Xing Ji, Heye Wang, Xiang Hou, Haichang Sun, Craig Billington, Lili Zhang, Xiaoye Wang, Ran Wang

**Affiliations:** ^1^College of Animal Science and Technology, Guangxi University, Nanning, Guangxi, China; ^2^Key Laboratory of Food Quality and Safety of Jiangsu Province-State Key Laboratory Breeding Base, Institute of Food Safety and Nutrition, Jiangsu Academy of Agricultural Sciences, Nanjing, China; ^3^College of Veterinary Medicine, Nanjing Agricultural University, Nanjing, China; ^4^Institute of Environmental Science and Research, Ilam, Christchurch, New Zealand

**Keywords:** *Staphylococcus aureus*, whole-genome sequencing, phylogenetic analysis, virulence factors, biofilm formation, invasion

## Abstract

*Staphylococcus aureus* is highly pathogenic and can cause disease in both humans and domestic animals. The aim of this study was to investigate the genomic epidemiology of *S. aureus* isolates from raw milk in Jiangsu Province, China, to identify predominant lineages and their associated genomic and phenotypic characteristics. In this study, we identified 117 *S. aureus* isolates collected from 1,062 samples in Jiangsu Province between 2021 and 2022. Based on whole-genome sequencing (WGS) data from 117 *S. aureus* isolates, molecular analyses indicated CC1-ST1 (26.50%, 31/117), CC97-ST97 (18.80%, 22/117), CC398-ST398 (10.26%, 12/117), CC8-ST630 (7.69%, 9/117) and CC59-ST59 (2.56%, 3/117) were the major lineages. The prevalence of *mecA*-positive strains was 11.11%. Four methicillin-resistant *S. aureus* (MRSA) lineages were found, including MRSA-ST59-t172 (*n* = 3), OS-MRSA-ST398-t011 (*n* = 1), MRSA-ST630-t2196 (*n* = 2) and OS-MRSA-ST630-t2196 (*n* = 7). Phenotypic resistance to penicillin (30.77%, 36/117), ciprofloxacin (17.09%, 20/117) and erythromycin (15.38%, 18/117) was observed which corresponded with resistance genotypes. All of the isolates could produce biofilms, and 38.46% (45/117) of isolates had invasion rates in mammary epithelial cells (MAC-T) of greater than 1%. Interestingly, most biofilm-producing and invading isolates harbored *ebp*-*icaA*-*icaB*-*icaC*-*icaR*-*clfA*-*clfB*-*fnbA*-*fnbB*-*sdrC*-*sdrD*-*sdrE*-*map*-*can* (27.35%, 32/117) and *ebp*-*icaA*-*icaB*-*icaC*-*icaD*-*icaR*-*clfA*-*clfB*-*fnbA*-*fnbB*-*sdrC*-*sdrD*-*sdrE*-*map* (33.33%, 39/117) adherence-associated gene patterns and belonged to lineages CC1 and CC97, respectively. Virulence factor assays showed that 47.01% of the isolates contained at least enterotoxin genes. Isolates harboring the immune evasion cluster (IEC) genes (*sea*, *sak*, *chp*, and *scn*) were predominantly categorized as STs 464, 398, and 59. IEC-positive ST398 and ST59 isolates contained a very high proportion of virulence genes located on prophages, whereas most IEC-negative ST398 clade isolates carried broad-spectrum drug resistance genes. Meanwhile, the IEC-positive ST398 clade showed a close genetic relationship with isolates from the pork supply chain and hospital surgical site infections. MRSA-ST59 strains showed the closest genetic relationship with an isolate from quick-frozen products. High-risk livestock-associated strains ST398 and MRSA-ST59 were detected in raw milk, indicating a potential public health risk of *S. aureus* transmission between livestock and humans. Our study highlights the necessity for *S. aureus* surveillance in the dairy industry.

## Introduction

Microbial contamination of raw milk is a public health risk factor that needs to be controlled in the production of dairy products. *Staphylococcus aureus* is one of the most common pathogens causing mastitis in dairy cattle, accounting for almost one-third of mastitis cases (Liu et al., 2017; Algammal et al., 2020). It has caused great economic losses in the global dairy industry due to reductions in milk yield and quality, culling of chronically infected cows and increasing treatment costs (Ren et al., 2020; Kotzamanidis et al., 2021). In addition, this pathogen can be transmitted to dairy products during the milking process and poses a potential hazard to humans. *S. aureus* infections in humans can be divided into community-associated SA (CA-SA), hospital-associated SA and livestock-associated SA (LA-SA). Zoonotic transmission of *S. aureus* between livestock and humans has been raised as a public health concern, especially for sequence types (ST) 398 and ST59 (Matuszewska et al., 2020; Yebra et al., 2022). In the last decade, *S. aureus* ST398 has been predominant in swine and humans, and occasionally reported from cattle in China (Cui et al., 2020; Li et al., 2022). Many cases have focused on human infections caused by LA-SA ST398 (Lienen et al., 2020; Zhao et al., 2020; Lienen et al., 2021). ST59 is known as the predominant ST type in CA-SA in eastern Asia (Chen et al., 2013). In recent years, its prevalence has gradually increased in China and it has been sporadically found in dairy farms (Frey et al., 2013; Chen C. et al., 2021).

Molecular typing methods have been widely implemented for the characterization of *S. aureus* epidemiology, including multiple-locus sequence typing (MLST), staphylococcal protein A (*spa*) typing and accessory gene regulator gene (*agr*) typing. However, these methods fail to reveal fine genetic relationships between strains. Whole-genome sequencing (WGS) has become the preferred method to understand phylogenetic relationships based on all genes in the core genome of strains (Naushad et al., 2016). In addition, WGS can predict potential antimicrobial resistance and virulence patterns based on complete gene banks of clinical isolates (Bakour et al., 2016).

Methicillin-resistant *S. aureus* (MRSA) is of significant concern due to its ability to resist multiple antibiotics, especially β-lactam antibiotics (Sekizuka et al., 2020). MRSA is typically resistant to oxacillin, but this is not always the case. Oxacillin-susceptible and *mecA*-positive (OS-MRSA) isolates have been described (Kong et al., 2015; Sabat et al., 2015; Liang et al., 2022). Compared to methicillin-susceptible *S. aureus* (MSSA), MRSA infections have caused significant healthcare system problems due to acute clinical outcomes (Chen H. et al., 2021; Rrapi et al., 2021).

The pathogenicity of *S. aureus* is mainly related to the expression of virulence factors. *S. aureus* may be capable of producing multiple types of toxins and staphylococcal enterotoxins are one of the leading causes of foodborne diseases worldwide (Boost et al., 2013). Toxic shock syndrome toxin-1 (*tsst*-*1*) can cause toxic shock syndrome by attenuating the host immune response. Panton-Valentine leucocidin (PVL), produced by the *lukF*-*PV* and *lukS*-*PV* genes, can target and lyse macrophages, leukocytes and monocytes and result in tissue necrosis (Yoong and Pier, 2012). In addition, *S. aureus* can evade human host immune responses by producing the immune response cluster (IEC), consisting of chemotaxis inhibitory protein gene (*chp*), staphylococcal complement inhibitor gene (*scn*), staphylokinase gene (*sak*), staphylococcal enterotoxin A gene (*sea*) and staphylococcal enterotoxin P gene (*sep*; Sieber et al., 2020). Thus, revealing the toxin gene profiles of *S. aureus* can improve our understanding of its pathogenicity.

The adoption of an intracellular lifestyle by *S. aureus* is important in its pathogenesis (Soe et al., 2021). *S. aureus* first colonizes the end of the teat, spreads into the breast through the milking process, then attaches to and enters the mammary epithelial cells (MAC-T; Naushad et al., 2020). Formation of biofilms is associated with evasion of host immune defenses, which helps *S. aureus* survive in the presence of antimicrobial and represents a more dangerous threat (Bazari et al., 2017). Several studies have demonstrated that some adherence genes are related to biofilm formation, such as intracellular adhesion locus gene cluster (*icaA*-*D*), gene *cna* encoding collagen binding proteins, *clfA*-*B* encoding clumping factors and gene *fnbA*-*B* encoding fibronectin binding proteins (Lister and Horswill, 2014; Wang W. et al., 2018).

The aim of this study was to systematically investigate the genetic diversity, antimicrobial resistance profiles and virulence characteristics of *S. aureus* strains in raw milk from dairy farms in Jiangsu Province, China. Phylogenetic analysis was carried out on the evolutionary dynamics of LA ST398 and MRSA-ST59 isolates. The data enabled tracking of the spread of *S. aureus* from raw milk and conclusions on potential public health risks.

## Materials and methods

### Isolation and identification

From 2021 to 2022, a total of 1,062 unique raw milk samples were collected from 90 dairy farms located in Jiangsu Province in China. Sample details are given in Supplementary Table S1, including the collection region and collection time. All samples were collected from healthy cows. The milk samples were stored in cold sterile containers and immediately transported to the laboratory. Milk samples were incubated in 7.5% NaCl broth for 24 h, then streaked onto chromogenic medium *S. aureus* agar (CHROMagar, Paris, France). Typical pink/purple colonies were picked for purification. *S. aureus* were confirmed by amplification of the species-specific thermonuclease gene *(nuc)* and VITEK 2 Compact Gram-positive identification card analysis (bioMérieux, Marcy-l’Étoile, France).

### Whole-genome sequencing and phylogenetic analysis

All confirmed *S. aureus* isolates were subjected to WGS. Genomic DNA from each isolate was extracted using a Bacteria DNA Extraction Kit (Magen, Guangzhou, China) according to the manufacturer’s instructions, then sequenced using an Illumina HiSeq X-Ten System (Illumina Inc., San Diego, CA, United States). Sequence reads were assembled using SPAdes^1^ and quality-filtered using Unicycler (version 0.4.8). Clonal complex (CC), MLST, *Staphylococcal* cassette chromosome *mec* (SCC*mec*) types and *spa* typing of all strains were conducted using online analysis tools^2^ (Ji et al., 2021). The *agr* allele types (I-IV) were assessed using multiplex PCR as previously described (Gilot et al., 2002) using primers shown in Supplementary Table S2. A Mash phylogenetic tree was constructed based on global mutation distances of the whole genome using Mash (v2.1) and visualized using iTOL.^3^ All known resistance and virulence genes were screened using the ResFinder and VirulenceFinder databases (>90% identity; Inouye et al., 2014). Prophages that carried IEC genes were identified from the assembled chromosomes of ST398 and ST59 isolates using the Prophage Hunter tool^4^ (Song et al., 2019). Prophage DNA sequences were run through the automatic annotation pipeline RAST,^5^ and a comparison of the genetic contex was generated using BLASTn and further visualized using Easyfig (v2.2.2; Sullivan et al., 2011). The genome assemblies of 117 isolates were deposited in GenBank and registered with the BioProject number PRJNA951802 (Supplementary Table S3).

### Antimicrobial susceptibility testing

Antimicrobial susceptibility testing was performed using the broth microdilution method according to Clinical and Laboratory Standards Institute 2020 guidelines for the following antimicrobial agents: tetracycline (TET), penicillin (PEN), oxacillin (OXA), ampicillin (AMP), vancomycin (VAN), gentamicin (GEN), ciprofloxacin (CIP), amikacin (AMI), clindamycin (CLI), erythromycin (ERY), chloramphenicol (CHL), moxifloxacin (MOX), streptomycin (STR), sulfamethoxazole (SUL), kanamycin (KAN) and rifampicin (RIF). *S. aureus* ATCC 29213 was used as a reference strain. All antimicrobial susceptibility testing assays were repeated at least three times. The MARI (Multiple antibiotic resistance index) for each isolate was calculated by the methods described by Krumperman (1983). An MARI >0.2 indicates the existence of an isolate from high-risk contaminated sources with frequent use antibiotics, whereas values ≤0.2 show that bacteria are from sources that have been exposed to less antibiotic usage (Woh et al., 2023).

### Biofilm formation assays

The ability of *S. aureus* to form biofilms was determined using the crystal violet primary staining method as previously described with some modifications (Ahmadrajabi et al., 2017). First, 20 μL of bacterial log phase culture was added to 180 μL Trypticase Soy Broth (TSB) supplemented with 1% glucose in 96-well flat-bottom microtiter plates. After incubation under aerobic conditions for 24 h at 37°C, wells were washed three times with 200 μL of sterile phosphate-buffered saline (PBS, pH 7.2) and drained by inversion. Subsequently, 200 μL of 95% ethanol was added to each well, and the plates were dried for 30 min. The adherent cells were stained with 200 μL of 0.1% crystal violet solution for 15 min, then washed twice with PBS. Bound crystal violet was dissolved by treatment with 200 μL of 33% acetic acid for 15 min, and the optical density (OD) at 570 nm was measured for stained bacteria and control wells. The experiment was performed in triplicate. As a negative control, 200 μL TSB + 1% glucose medium was used to determine the background OD [cut-off value (ODc) = average OD of negative control +3× standard deviation (SD) of negative control] (Pereyra et al., 2016). OD > 4× ODc indicated strong biofilm producers, 2× ODc < OD ≤ 4× ODc indicated moderate biofilm producers and ODc < OD ≤ 2× ODc and OD ≤ ODc indicated weak and non-biofilm producers, respectively.

### Invasion assays

Bovine mammary epithelial cells were used for *in vitro* bacterial internalization assays as previously described (Bardiau et al., 2014). The MAC-T cells were grown in Dulbecco’s modified Eagle’s medium supplemented with 10% fetal calf serum (Sigma-Aldrich, St. Louis, MO, United States) without antibiotics. Prior to each experiment, MAC-T cells were seeded at 2 × 10^5^ cells/well in 24-well culture plates and incubated for 12 h at 37°C in 5% CO_2_. Approximately 4 h prior to the invasion experiments, cells were washed twice with PBS and incubated with invasion medium (growth medium without antibiotics containing 1% fetal calf serum). The isolates were incubated for 6 h at 37°C to the logarithmic growth phase and resuspended in Dulbecco’s modified Eagle’s medium without serum. Then, the cells were infected with bacteria (multiplicity of infection = 10:1) and co-incubated for 2 h at 37°C. Subsequently, the supernatant was aspirated and discarded and the plates were washed three times with sterile PBS and incubated with 10 μg/mL lysostaphin and 200 μg/mL gentamicin medium for 2 h to remove extracellular bacteria. After cells were dissociated with 200 μL trypsin–EDTA, MAC-T cells were lysed with 0.05% Triton X-100. Bacterial colony-forming units were enumerated using the plate counting method. These invasion assays were performed in triplicate.

### Comparative genomic analysis of domestic ST398 and ST59 isolates

To clarify the genetic relatedness of ST398 and ST59 in this study and domestic isolates, 67 published WGS datasets for the ST398 strain and 78 published WGS datasets for the ST59 strain were downloaded from the GenBank database and included for comparative genomic analysis (Supplementary Tables S4, S5). Mash phylogenetic trees of different species were generated based on global mutation distances of the whole genome using Mash (v2.1) and further visualized using iTOL.

### Statistical analyses

Statistical analysis was performed using GraphPad Prism software (Version 5.01; GraphPad, San Diego, CA, United States).

## Results

### Isolation of *Staphylococcus aureus* from milk and molecular analyses

**Figure 1:**
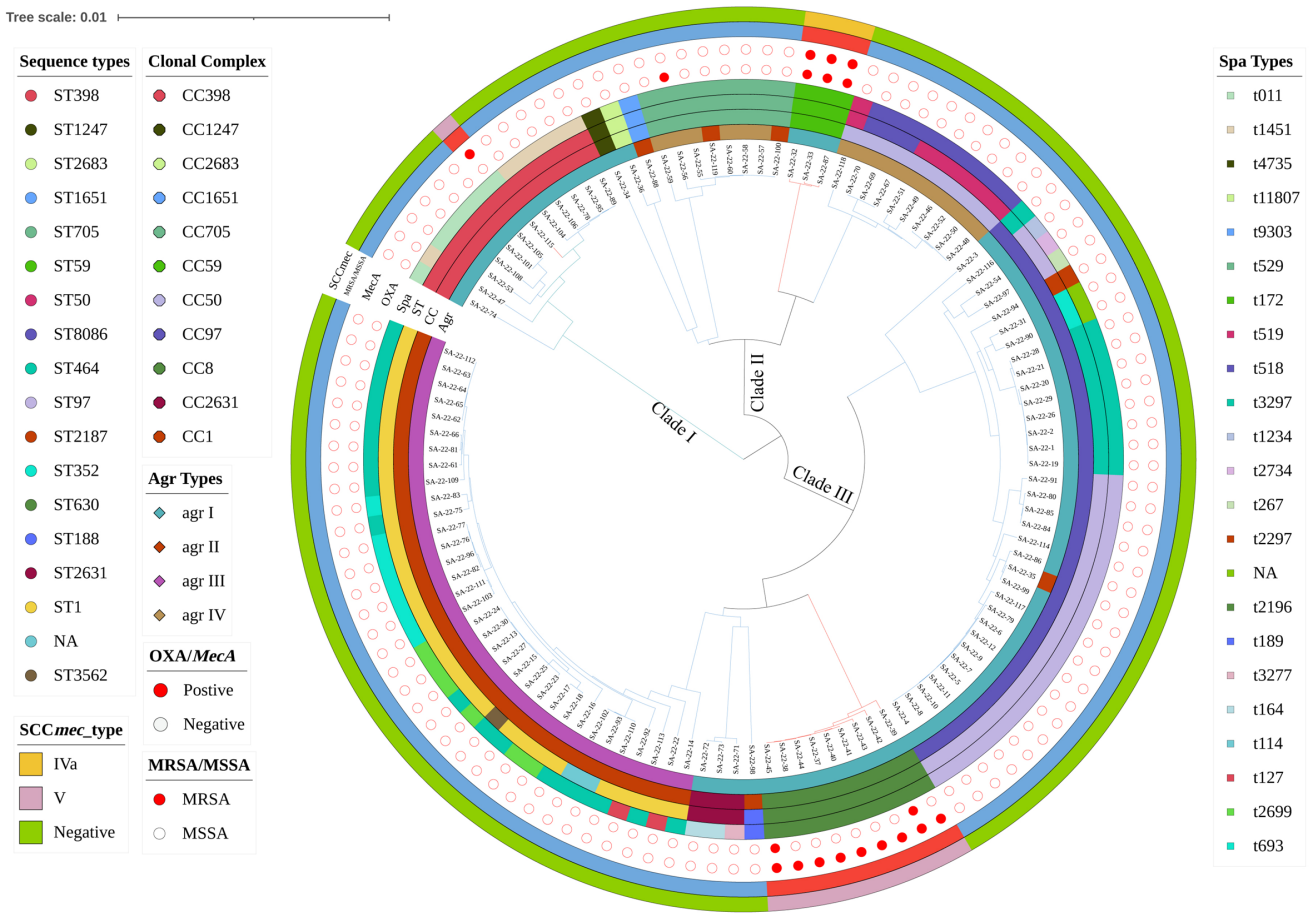
Phylogenetic analysis of *Staphylococcus aureus* isolates. Showing distribution of clonal complexes (CCs), sequence types (STs), polymorphism of protein A gene (*spa* typing), accessory gene regulator gene (*agr* typing) and carrying of *mecA*. Mash phylogenetic trees were constructed based on the global mutation distance of the whole genome using Mash (v2.1) and overlayed with information regarding CCs, STs, *spa* typing, and *agr* typing using iTOL.

In the 1,062 raw milk samples collected, 117 *S. aureus* isolates were confirmed, and of these 104 (88.89%) were MSSA and 13 (11.11%) MRSA (Figure 1). Whole genome sequencing revealed the isolates grouped into three major phylogenetic clades, with 18 distinct STs that belonged to 11 CCs. The most prevalent strains belonged to CC1 (29.06%, 34/117) and CC97 (29.06%, 34/117). Analysis of MLST data indicated that CC1-ST1 was most frequent (26.50%, 31/117), followed by CC97-ST97 (18.80%; 22/117), CC398-ST398 (10.26%, 12/117), CC8-ST630 (7.69%, 9/117) and CC59-ST59 (2.56%, 3/117).

There were 23 distinct *spa* types found, with t2734 (17.09%, 20/117) and t114 (16.24%, 19/117) the most frequently isolated (Figure 1; Supplementary Table S2). Furthermore, *agr* types were identified in 117 isolates by multiplex PCR. As shown in Figure 1, *agr*I was most prevalent (53.85%, 63/117), followed by *agr*III (29.06%, 34/117), *agr*IV (13.68%, 16/117) and *agr*II (3.42%, 4/117). SCC*mec* typing revealed only two types (types IV and V) among 13 MRSA isolates. Type V was the most predominant type and was found in ten isolates (ST630 and ST398; 76.92%, 10/13). Type IV was found in three ST59 isolates (23.08%, 3/13).

MRSA lineages were investigated by combining *agr* type, MLST, *spa* and SCC*mec*. Four MRSA lineages were found, including MRSA-*agr*I-ST59-t172-SCC*mec*IVa (*n* = 3), OS-MRSA-*agr*I-ST398-t011-SCC*mec*Va (*n* = 1), MRSA-*agr*I-ST630-t2196-SCC*mec*Va (*n* = 2) and OS-MRSA-*agr*I-ST630-t2196-SCC*mec*Va (*n* = 7; Figure 1).

### Antimicrobial susceptibility testing and antimicrobial resistance gene analysis

**Figure 2:**
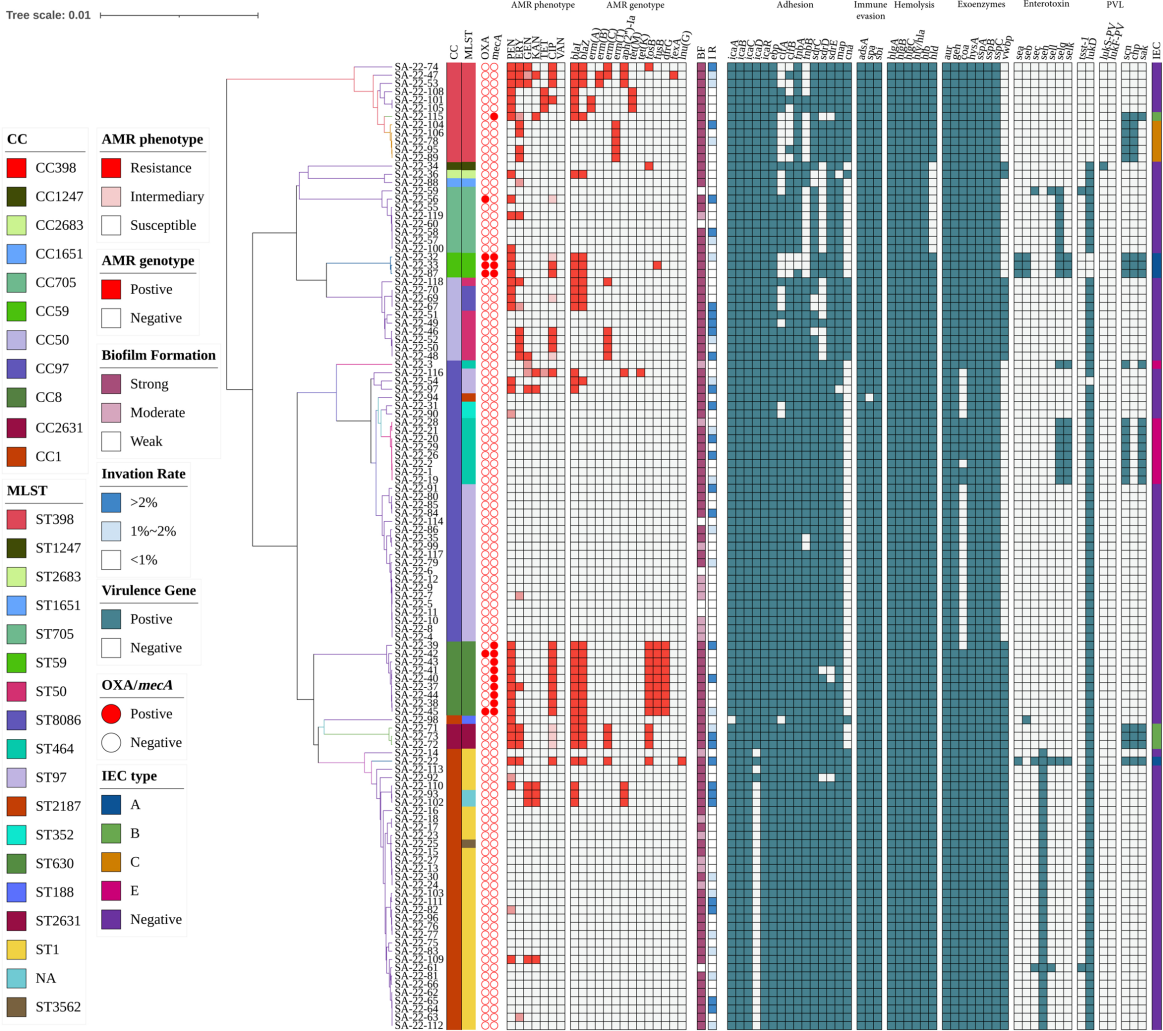
Heatmap of antibiotic resistance phenotypes and genotypes, biofilm formation, invasion rate of mammary epithelial cells (MAC-T) and carriage of virulence genes for 117 *S. aureus* isolates. The distributions of antimicrobial genes, antibiotic susceptibility profiles and carriage of virulence genes are plotted against the core genome phylogeny of the *S. aureus* isolates. BF, biofilm formation; IR, invation rate.

**Figure 3:**
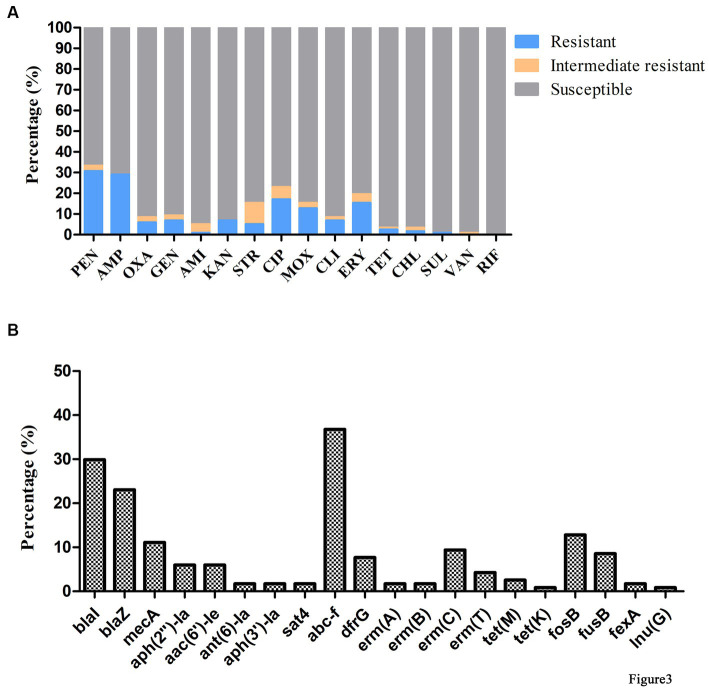
Antimicrobial phenotype and genotype results of *S. aureus* isolated from raw milk samples. **(A)** Antimicrobial susceptibility tests of 117 *S. aureus* strains isolated from raw milk samples. AMI, amikacin; AMP, ampicillin; CHL, chloramphenicol; CIP, ciprofloxacin; CLI, clindamycin; ERY, erythromycin; GEN, gentamicin; KAN, kanamycin; MOX, moxifloxacin; OXA, oxacillin; PEN, penicillin; RIF, rifampicin; SRT, streptomycin; SUL, sulfamethoxazole; TET, tetracycline; VAN, vancomycin. **(B)** Detection of antimicrobial resistance genes in 117 *S. aureus* strains isolated from raw milk samples.

Antimicrobial susceptibility testing and antimicrobial resistance gene analysis of the 117 isolates showed most *S. aureus* isolates were susceptible to frequently-used veterinary antimicrobial agents (Figure 2; Supplementary Table S3). The overall resistance rates were as follows: PEN, 30.77% (36/117); AMP, 29.06% (34/117); CIP, 17.09% (20/117); ERY, 15.38% (18/117); and MOX, 12.82% (15/117; Figure 3A; Supplementary Table S3). Supplementary Table S3 showed that 19.6% of isolates had an MARI >0.2 and 80.34% of isolates had an MARI ≤0.2 (Supplementary Table S3). The *mecA* gene was detected in 13 isolates, including five isolates that were resistant to OXA (MRSA) and eight isolates that were sensitive to OXA (OS-MRSA; Figures 1, 2). The resistance genes *blaI*, *blaZ*, *fosB*, *erm(C)*, *fusB*, *dfrG*, *aph(2″)*-*Ia*, *tet(M)*, *fexA* and *lnu(G)* had prevalence rates of 29.91% (35/117), 23.08% (27/117), 12.82% (15/117), 9.40% (11/117), 8.55% (10/117), 7.69% (9/117), 5.98% (7/117), 2.56% (3/117), 1.71% (2/117) and 0.85% (1/117), respectively (Figure 3B; Supplementary Table S3). The antimicrobial susceptibility test results confirm the wide distribution of antimicrobial resistance genes observed in the *S. aureus* isolates (Figure 2; Supplementary Table S3). The *blaI* gene, which confers β-lactam resistance, was supported by phenotypic resistance results for ST398, ST630 and ST59 isolates (Figure 2). Three ST398 isolates were resistant to TET, which was consistent with the positivity rate of *tet(M)* (Figure 2).

### Prevalence and distribution of virulence genes

Analysis of virulence genes in the 117 isolates showed the intercellular adhesion protein genes (*icaB*-*C*, *icaR*), surface elastin binding protein gene (*ebp*), serine protease genes (*sspA*-*C*), hemolysin genes (*hlgA*-*C*, *hly/hla*, and *hlb*) were present in all isolates (Figure 2). Seven different staphylococcal enterotoxin gene profiles were detected in various combinations in 47.01% (55/117) of *S. aureus* isolates (Table 1). The enterotoxin gene *seh* was found in most isolates (29.06%, 34/117), all of which belonged to CC1 (Figure 2; Table 1). The PVL gene *lukS*-*PV* was detected in an isolate of ST1247. Two strains carrying the *tsst*-*1* gene were detected in the *sec*-*seh*-*sell* and *sec*-*sell*-*selq* enterotoxins clusters (ST1 and ST705). The distribution of some enterotoxin genes was correlated to ST, for example, *icaA* was detected in all isolates except ST188, and most CC1 isolates were negative for *icaD* (Figure 2).

**Table 1:** Enterotoxin gene profiles of *Staphylococcus aureus* isolates (*n* = 117).

Toxin gene profiles	Number (%) of isolates	MLST	IEC type
*seb*	1(0.85%)	ST188	/
*seh*	29(24.79%)	ST1	/
	1(0.85%)	ST352	/
	2(1.70%)	NA	/
*selq*	7(5.93%)	ST705	/
*selq-selk*	9(7.63%)	ST464	E
*sec-seh-sell*	1(0.85%)	ST1	/
*sec-sell-selq*	1(0.85%)	ST705	/
*sea- seb- selq-selk*	3(2.54%)	ST59	A
*sea-sec-seh-sell-selq-selk*	1(0.85%)	ST1	A
total	55(47.01%)		

**Figure 4:**
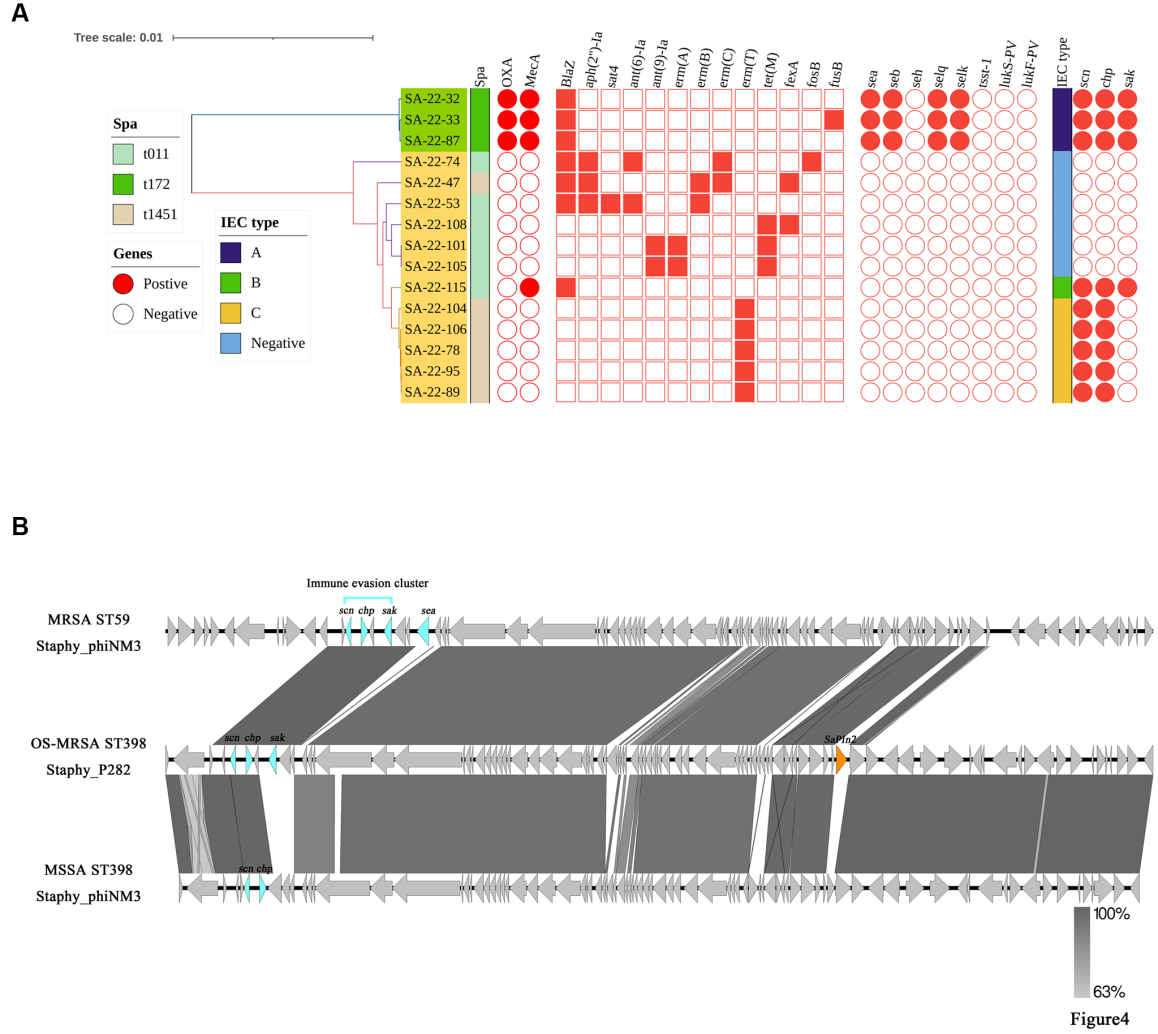
Distributions of virulence and antimicrobial genes, and genetic structure of prophages carrying immune evasion cluster (IEC) genes in ST398 and ST59 isolates. **(A)** The main virulence and antimicrobial genes in ST398 and ST59 isolates. The Mash phylogenetic tree was constructed based on global mutation distances of the whole genome using Mash (v2.1). **(B)** Genetic structure of prophages carrying immune evasion cluster genes identified from ST59 and ST398 isolates in this study.

IEC genes were found in 18.80% (*n* = 22) of isolates (Table 2) and were divided into four types according to analysis of five genes of the IEC system: A (*sea*-*sak*-*chp*-*scn*, *n* = 4), B (*sak*-*chp*-*scn*, *n*= 4), C (*chp*-*scn*, *n*= 5) and E (*sak*-*scn*, *n*= 9). Among the IEC-positive strains, the predominant IEC type was type E (9/22) distributed in ST464. Furthermore, all MRSA ST59 clones belonged to IEC type A, one OS-MRSA ST398 clone belonged to IEC type B and five ST398 clones lacked the *sak* gene and belonged to IEC type C (Table 2; Figure 4A).

**Table 2:** Summary of IEC types of *Staphylococcus aureus* isolates (*n* = 117).

IEC Type	IEC genes composition	Number of isolates		Total number of isolates
		MSSA (*n* = 104)	MRSA (*n* = 13)	
A	*sea, sak, chp, scn*	1(ST1)	3(ST59)	4
B	*sak, chp, scn*	3(ST2631)	1(ST398)	4
C	*chp, scn*	5(ST398)	0	5
D	*sea, sak, scn*	0	0	0
E	*sak, scn*	9(ST464)	0	9

### Formation of biofilms and invasion of MAC-T cells

In this study, all *S. aureus* isolates were able to form biofilms, with 80.34% (94/117) able to form strong biofilms and 5.98% (7/117) weak and 13.68% (16/117) moderate biofilm producers (Figure 2). All ST59 and ST398 isolates showed strong biofilm formation ability.

MAC-T invasion assays showed that 72 isolates (61.54%) had an invasion rate of less than 1%, 27 (23.08%) had an invasion rate greater than 2% of the initial inoculum, and 18 isolates (15.38%) had an invasion rate between 1 and 2% (Figure 2). In addition, 41.67% (5/12) of ST398 isolates and 33.33% (1/3) of ST59 isolates had a MAC-T invasion rate greater than 1% (Figure 2). Moreover, we found that all strains with a MAC-T invasion rate greater than 1% formed strong biofilms (Figure 2).

The relationship between the prevalence of adherence-associated genes, biofilm formation ability and ability to invade MAC-T cells is further analyzed in Table 3. Twenty-seven different gene patterns were observed; the most prevalent gene patterns were *ebp*-*icaA*-*icaB*-*icaC*-*icaR*-*clfA*-*clfB*-*fnbA*-*fnbB*-*sdrC*-*sdrD*-*sdrE*-*map*-*can* (27.35%, 32/117) and *ebp*-*icaA*-*icaB*-*icaC*-*icaD*-*icaR*-*clfA*-*clfB*-*fnbA*-*fnbB*-*sdrC*-*sdrD*-*sdrE*-*map* (33.33%, 39/117). Of these isolates, 73.24% (52/71) showed strong biofilm formation ability, and 38.03% (27/71) showed a MAC-T invasion rate greater than 1%. These gene patterns were mainly associated with CCs; the *ebp*-*icaA*-*icaB*-*icaC*-*icaR*-*clfA*-*clfB*-*fnbA*-*fnbB*-*sdrC*-*sdrD*-*sdrE*-*map*-*can* gene pattern mainly belonged to CC1, whereas the *ebp*-*icaA*-*icaB*-*icaC*-*icaD*-*icaR*-*clfA*-*clfB*-*fnbA*-*fnbB*-*sdrC*-*sdrD*-*sdrE*-*map* gene pattern belonged to CC97 (Figure 2).

**Table 3:** Prevalence of biofilm-related gene patterns and their associations with biofilm production and ability to invade bovine mammary epithelial cells for 117 *Staphylococcus aureus* isolates.

Adhesion related genes patterns	Biofilm production			Invasion rate		
	Strong	Moderate	Weak	<1%	1 ~ 2%	>2%
*ebp-icaA-icaB-icaC-icaD-icaR-clfB-fnbA-sdrC-sdrD-sdrE-map-can*	2	0	0	1	1	0
*ebp-icaA-icaB-icaC-icaD-icaR-fnbA-sdrC-sdrD-sdrE-map-can*	6	0	0	2	2	2
*ebp-icaA-icaB-icaC-icaD-icaR-clfA-fnbA-sdrC-sdrD-sdrE-map-can*	1	0	0	1	0	0
*ebp-icaA-icaB-icaC-icaD-icaR-clfA-clfB-fnbA-fnbB-sdrC-sdrD-sdrE-map-can*	2	0	0	1	0	1
*ebp-icaA-icaB-icaC-icaR-clfA-clfB-fnbA-fnbB-sdrC-sdrD-sdrE-map-can*	25	6	1	19	6	7
*ebp-icaA-icaB-icaC-icaD-icaR-clfA-fnbA-fnbB-sdrC-sdrD-sdrE-map-can*	1	0	0	1	0	0
*ebp-icaA-icaB-icaC-icaD-icaR-fnbA-sdrC-map-can*	1	0	0	1	0	0
*ebp-icaA-icaB-icaC-icaD-icaR-clfB-fnbA-fnbB-sdrE-can*	1	0	0	1	0	0
*ebp-icaB-icaC-icaD-icaR-clfB-fnbA-fnbB-sdrC-sdrD-sdrE-map-can*	1	0	0	1	0	0
*ebp-icaA-icaB-icaC-icaD-icaR-clfA-clfB-fnbA-fnbB-sdrC-map-can*	1	0	0	1	0	0
*ebp-icaA-icaB-icaC-icaD-icaR-clfA-clfB-fnbA-fnbB-sdrE-map-can*	1	0	0	0	0	1
*ebp-icaA-icaB-icaC-icaD-icaR-clfA-clfB-fnbA-fnbB-sdrC-sdrE-map-can*	1	0	0	0	0	1
*ebp-icaA-icaB-icaC-icaD-icaR-clfB-fnbA-fnbB-sdrC-sdrD-sdrE-map-can*	1	0	0	0	0	1
*ebp-icaA-icaB-icaC-icaD-icaR-clfA-clfB-fnbA-fnbB-sdrC-sdrE-map-can*	4	0	0	2	1	1
*ebp-icaA-icaB-icaC-icaD-icaR-clfB-fnbA-fnbB-sdrE-map-can*	3	0	0	2	0	1
*ebp-icaA-icaB-icaC-icaD-icaR-clfB-fnbA-fnbB-sdrC-sdrE-map-can*	1	0	0	1	0	0
*ebp-icaA-icaB-icaC-icaD-icaR-clfA-clfB-fnbA-fnbB-sdrC-sdrD-sdrE-map*	27	8	4	25	7	7
*ebp-icaA-icaB-icaC-icaD-icaR-clfA-clfB-fnbA-sdrC-sdrD-sdrE-map*	1	1	0	2	0	0
*ebp-icaA-icaB-icaC-icaD-icaR-clfA-clfB-fnbA-fnbB-sdrC-sdrD-map*	1	0	0	0	0	1
*ebp-icaA-icaB-icaC-icaD-icaR-clfA-clfB-fnbA-fnbB-sdrC-map*	1	0	0	1	0	0
*ebp-icaA-icaB-icaC-icaD-icaR-sdrC-sdrD-sdrE-map*	2	0	0	1	1	0
*ebp-icaA-icaB-icaC-icaD-icaR-fnbA-sdrC-sdrD-sdrE-map*	1	0	0	1	0	0
*ebp-icaA-icaB-icaC-icaD-icaR-clfA-clfB-fnbA-fnbB-sdrC-sdrE-map*	1	0	0	1	0	0
*ebp-icaA-icaB-icaC-icaD-icaR-clfA-clfB-fnbA-sdrC-sdrE-map*	5	1	1	4	1	2
*ebp-icaA-icaB-icaC-icaD-icaR-clfB-fnbA-sdrC-sdrE-map*	0	0	1	1	0	0
*ebp-icaA-icaB-icaC-icaD-icaR-clfB-fnbA-fnbB-sdrC-sdrD-sdrE-map*	2	0	0	1	0	1
*ebp-icaA-icaB-icaC-icaD-icaR-clfA-clfB-fnbA-fnbB-sdrC-sdrD-sdrE*	1	0	0	0	0	1

### Analysis of antimicrobial resistance and virulence genes between *Staphylococcus aureus* ST59 and ST398

The core genome, resistance and virulence genes of the potentially zoonotic ST398 and ST59 isolates were compared (Figure 4A). Twelve ST398 isolates were divided into two *spa* types (t011 and t1451). Three ST59 isolates belonged to t172. The phylogenetic tree showed that the ST398 isolates were divided into an IEC-positive clade and IEC-negative clade. The IEC-positive OS-MRSA ST398 strain only carried β-lactamase resistance genes, whereas most IEC-negative ST398 isolates carried broad spectrum antimicrobial resistance genes (Figure 4A).

Compared with ST59-t172, ST398-t011 carried more resistance genes and fewer virulence genes. Enterotoxin genes, including *sea*, *seb*, *selq* and *selk* were detected in all ST59-t011 isolates but were absent from all ST398 isolates (Figure 4A). We further evaluated the different prophages carrying IEC genes in the ST59 and ST398 strains. We found that prophage Staphy_phiNM3 carried IEC genes in MRSA ST59-t172 isolates and MSSA ST398-t1451 isolates, whereas prophage Staphy_P282 carried IEC genes in OS-MRSA ST398-t011 isolates (Figure 4B). Other virulence genes, such as the pathogenicity island SaPIn2 and *sea,* were also found in prophages Staphy_P282 and Staphy_phiNM3 from OS-MRSA ST398-t011 and MRSA ST59-t172 (Figure 4B). The prophage Staphy_phiNM3 in MSSA ST398-t1451 carried IEC genes only including *scn* and *chp* (Figure 4B). The genome of prophage Staphy_P282 from OS-MRSA ST398-t011 showed 99.80% nucleotide identity with that of Staphy_phiNM3 MSSA from ST398-t1451 (Figure 4B). The genomes of prophage Staphy_phiNM3 showed 98.79% nucleotide identity between OS-MRSA ST59-t172 and MSSA ST398-t1451 isolates (Figure 4B).

### Phylogenetic construction of *Staphylococcus aureus* ST59 and ST398 clonotypes

**Figure 5:**
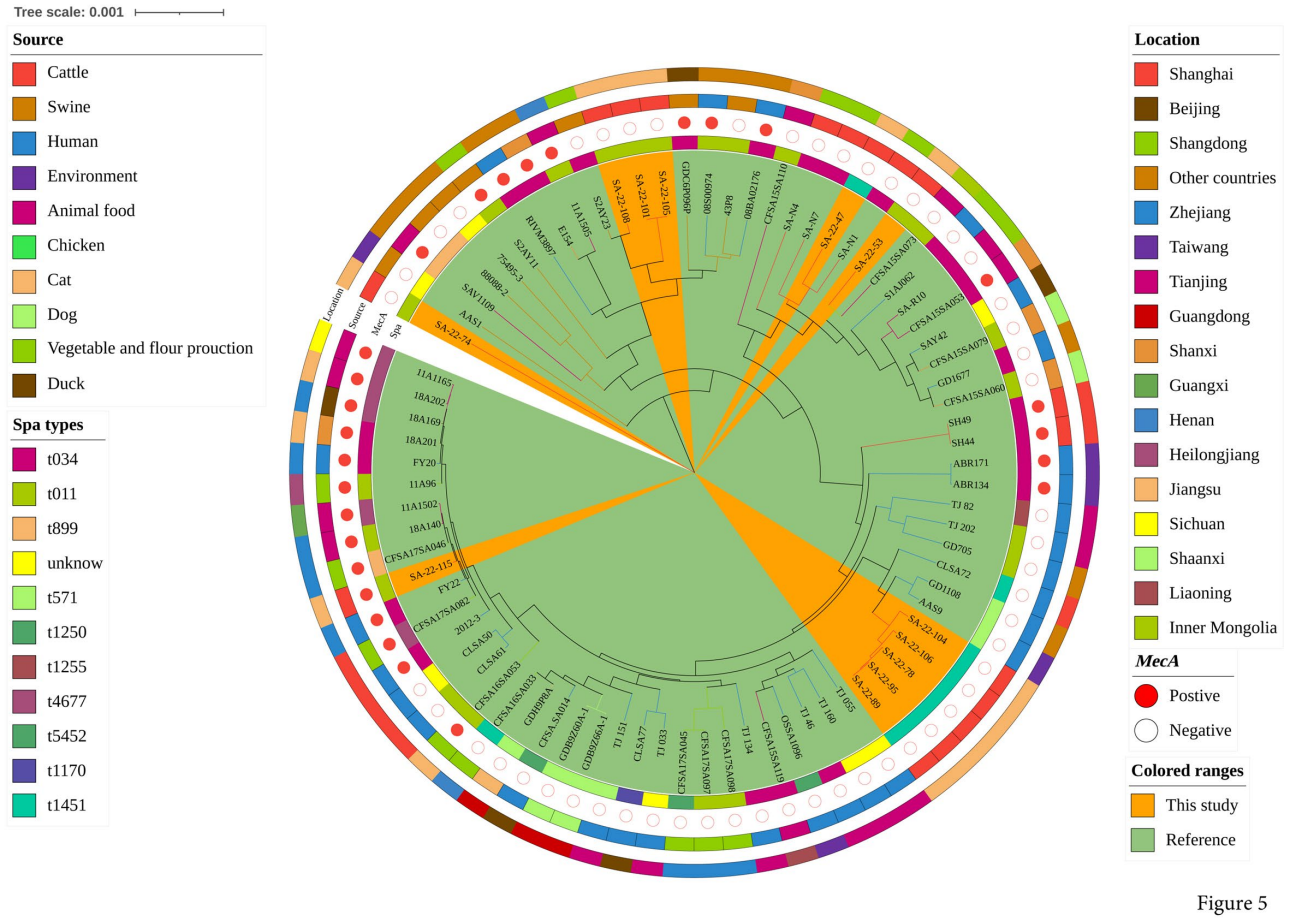
Phylogenetic analysis of ST398 isolates. Classification of location, source, polymorphism of protein A gene (*spa* typing) and presence of *mecA* for 79 domestic ST398 isolates. Strains from this study and publicly-available reference genomes are indicated by leaf color. The Mash phylogenetic tree was constructed based on global mutation distances of the whole genome using Mash (v2.1) and cleaned and overlayed using iTOL.

**Figure 6:**
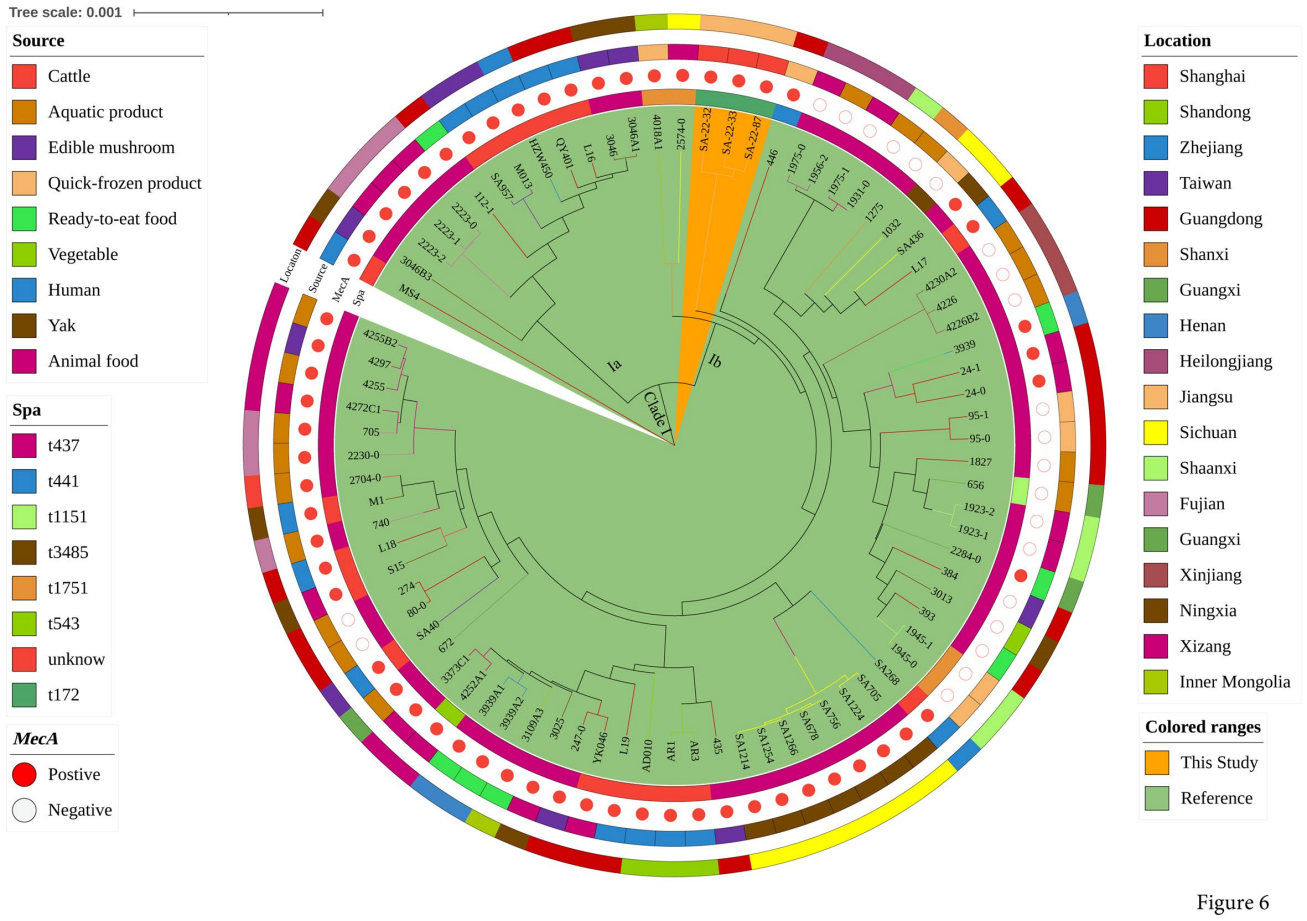
Phylogenetic analysis of ST59 isolates. Classification of location, source, polymorphism of protein A gene (*spa* typing) and presence of *mecA* for 81 domestic ST59 isolates. Isolates from this study and publicly-available reference genomes are indicated by leaf color. The Mash phylogenetic tree was constructed based on the global mutation distance of the whole genome using Mash (v2.1) and cleaned and overlayed using iTOL.

To explore the evolution of ST398 isolates, we constructed a phylogenetic tree with our 12 ST398 isolates and 67 ST398 isolates from the NCBI database, (including 11 ST398 isolates from other countries; Supplementary Table S3). The 12 ST398 isolates from this study could be divided into different clades, with the OS-MRSA strain SA-22-115 showing a close genetic relationship with the isolate MRSA FY22 (NXFU00000000) from a surgical site infection (Figure 5). MSSA strains SA-22-78, 89, 95, 104 and 106 were closely related to the MSSA isolate AAS9 (NZ_VKAP00000000) from a pork processing worker. MSSA strain SA-22-47 was closely related to the dairy MSSA isolate SA-N1 (JAHVAQ000000000) originating from bulk tank milk. For the ST59 isolates, two clades were distinguished (Figure 6). All of the strains from clade Ia were MRSA, and our three MRSA ST59 isolates resided in clade Ib, showing a close genetic relationship with strain 446 (WKZQ00000000) from quick-frozen products (Figure 6; Supplementary Table S4).

## Discussion

*Staphylococcus aureus* is a pathogenic microorganism that can be transmitted by both humans and farm animals and its control is important for maintaining public health. In this study, the prevalence of *S. aureus* in raw milk samples sourced from Jiangsu Province was 11.01% (117/1062), which is similar to a recent report in China (Zhang et al., 2022). Epidemiological investigation of these *S. aureus* strains was undertaken by comprehensive analyses incorporating genotypic and phenotypic data to examine population structure, antimicrobial resistance, distribution of virulence genes, biofilm formation ability, invasion of MAC-T cells and intracellular survival. These data provide in-depth insights into *S. aureus* clone prevalence in raw milk in China and will strengthen control strategies.

The 117 *S. aureus* isolates were grouped into 11 CCs, including CC1, CC97, CC398, CC59, CC50, CC8 and CC705. CC1 and CC97 were the most common in this study. Additionally, we found novel sequence type ST8086, which was identified as a single-locus variant of ST50. The literature indicates CC97, CC50, CC705 and CC8 are predominant in bovine milk (Liu et al., 2020; Miyazawa et al., 2022), and CC1, CC59 are able to cause disease in multiple host species (Côrtes et al., 2018; Wang Y. et al., 2018; Côrtes et al., 2021). CC59 is rarely linked to LA-SA infections, has mostly been reported in CA-SA infections (Chen et al., 2012; Wang X. et al., 2018) and is predominant among human infections (Chen H. et al., 2021). Compared with our previous study in 2016, the *agr* type of *S. aureus* isolates detected in milk has changed in Jiangsu Province, but *agr*I is still the most common type (Zhang et al., 2016).

In the present study, two-thirds of isolates (84/117) were susceptible to most of the antimicrobials tested and lacked the associated antimicrobial resistance genes, which is similar to a report that 31.3% (30/96) of *S. aureus* isolates were resistant to at least one antimicrobial agent. However, much lower than another report that 100% (298/298) of *S. aureus* isolates were resistant to two antimicrobials (Wang W. et al., 2018; Zhang et al., 2022). The highest rates of antimicrobial resistance were against PEN and AMP, followed by CIP and MOX, which is generally in accordance with previous reports (Wang W. et al., 2018). OS-MRSA represented 61.54% (8/13) of MRSA isolates. However, conventional susceptibility testing methods are difficult as exposure to β-lactam antibiotics can cause OXA resistance to develop, which further increases the difficulty of prevention (Saeed et al., 2014). We found a high correlation between resistance phenotypes and resistance genotypes: 100% (3/3) of TET resistant isolates carried *tet*, 83.33% (15/18) of ERY resistant had *erm* and 75% (27/36) of PEN resistant carried *blaZ*. Resistance to PEN, CIP and ERY has gradually increased in *S. aureus* isolated (Martini et al., 2017; Kaczorek-Łukowska et al., 2022), indicating these antibiotics need to be carefully managed.

Biofilms help *S. aureus* tolerate the effects of antimicrobial agents and contributes to their long persistence in stressful environments, increasing the difficulty of elimination from both foods and equipment. In addition, biofilm formation by *S. aureus* correlates with the source of clinical infection (Kwiecinski et al., 2019). In this study, the *vitro* biofilm formation assay showed that all our isolates could form biofilms, and at least 80% of isolates showed the ability to produce strong biofilms. A previous study by Shen et al. (2021) showed that the biofilm formation abilities of raw milk isolates were stronger than that of human origin strains when milk was present. Pereyra et al. (2016) found that *icaA* and *icaD* genes were detected in all bovine milk isolates, whereas the biofilm-related genes *icaB* and *icaC* were detected in all strains in this study. In addition, we found that strains with the ability to invade MAC-T cells (>1%) formed strong biofilms. This suggests need to make further efforts to prevent biofilm formation and the establishment of biofilm-forming *S. aureus* in the food supply chain environment.

Virulence genes can play an important role in the pathogenicity of *S. aureus* and, in particular, their enterotoxins cause sporadic food-poisoning incidents that seriously threaten public health. Staphylococcal enterotoxins are heat resistant and the enterotoxins still have emetic activity even after removing the *S. aureus* from heated food (Hennekinne et al., 2012). Many studies have shown that the *seb* and *sed* enterotoxin genes are mainly detected among isolates from raw milk (Song et al., 2015; Tarekgne et al., 2016). In the current study, four of the isolates were positive for the *seb* gene, and all of the isolates were negative for the *sed* gene. Overall, 47.01% (55/117) of *S. aureus* isolates carried at least one enterotoxin gene, and *seh* (29.06%, 34/117) was the most frequently detected. Enterotoxin SEH has been found to be an increasing causative agent of food poisoning (Jørgensen et al., 2005).

Prophages are one of the primary vehicles for the horizontal transfer of virulence genes and are a key factor in the emergence of new virulent lineages. Numerous studies have focused on encoding for PVL and the IEC of *S. aureus* prophages (Kahánková et al., 2010; Xia and Wolz, 2014). Except for immune escape genes, the *sea* gene is also present in the genome of MRSA ST59 in a prophage region of Staphy_phiNM3 type. However, similar to previous studies, *S. aureus* prophages rarely carried antibiotic resistance genes (Ene et al., 2021).

*S. aureus* ST398 isolates represent two main subpopulations: the human-adapted clade (CA), and the livestock-associated clade (LA) that has lost the prophage carrying IEC genes. Almost all reported human-adapted ST398 isolates are MSSA and harbor the IEC system (Mama et al., 2021). In this study, 12 ST398 strains were identified, including IEC positive (*n* = 6) and IEC negative (*n* = 6). Five MSSA ST398 strains were IEC type C and isolate OS-MRSA-ST398-t011-SCC*mec*Va was IEC type B. Zou et al. (2022b) found ST398 isolates from pig and farm worker samples lacked IEC genes in comparison to ST398 isolates from patients. Moreover, 18.8% (22/117) of all isolates in this study were found to contain at least one IEC gene, and all contained the *scn* gene, which differs from previous reports that the absence of the *scn* gene is considered to be a marker for livestock-derived *S. aureus* strains (Cuny et al., 2015; Wardyn et al., 2015). All our ST398 strains lacked the *lukS-PV* and *lukF-PV* genes encoding the Panton-Valentine leucocidin cytotoxin, which is similar to a report of ST398 strains from pigs in Qinghai, China (Cui et al., 2022). All three MRSA-ST59-t172-SCC*mec*IVa strains in this study belonged to IEC type A and carried enterotoxin genes *sea*-*seb*-*selq*-*selk*, which contrasts to reports that 90.0% of ST59 isolates are IEC type B (Pang et al., 2020; McClure et al., 2021; Zou et al., 2022a).

In this study, OS-MRSA ST398 strain SA-22-115 was closely related to a MRSA ST398 isolate from a surgical site infection and five MSSA ST398 strains (SA-22-78, 89, 95, 104 and 106) were closely related to a ST398 strain from the pork supply chain. This may indicate that LA-MRSA ST398 originated from human MSSA-ST398 and then transmitted to livestock where it acquired methicillin resistance. This transmission and acquisition route is consistent with that reported by Yebra et al. (2022). Three MRSA ST59 isolates showed a close genetic relationship with MRSA isolate from quick-frozen products (frozen dumplings/steamed stuffed buns and frozen meat; Pang et al., 2020). This indicates that MRSA ST59 may have originated from humans and that the emergence of MRSA ST59 in raw milk samples does not exclude human dissemination to bovines but does further increase the risk of human infection, which should be the target of ongoing surveillance in dairy supply chain. Thus, further research should be conducted to determine the potential transmission risk of ST398 and ST59 for food outbreaks.

However, our understating of evolutionary epidemiology of *S. aureus* in raw milk is currently limited due to sampling and geographic constraints. Sources of sampling need to be further expanded and monitoring needs to be continuously implemented to overcome these hurdles and enable the development of improved prevention strategies for controlling *S. aureus* dissemination in the dairy food chain.

## Conclusion

We examined *S. aureus* isolated from raw dairy milk in Jiangsu Province in China for population structure, distribution of resistance and virulence genes, biofilm formation and invasion of bovine mammary epithelial cells. Our data indicate that these *S. aureus* isolates carry a high number of enterotoxin genes and have high biofilm formation capability. Furthermore, high-risk LA ST398 and MRSA ST59 lineages were detected at dairy farms, indicating a potential public health risk of transmission of *S. aureus* between livestock and humans. Thus, awareness should be raised to strengthen the surveillance and control of *S. aureus* in dairy supply chain.

## Data availability statement

The datasets presented in this study can be found in online repositories. The names of the repository/repositories and accession number(s) can be found at: https://www.ncbi.nlm.nih.gov/genbank/, PRJNA951802.

## Ethics statement

This study was performed following the Chinese guidelines for animal welfare, and the animal protocol was approved by the Animal Welfare Committee of Jiangsu Academy of Agricultural Sciences (permit number SYXK [Su] 2016-0020). The study was conducted in accordance with the local legislation and institutional requirements.

## Author contributions

HL: Data curation, Methodology, Formal analysis, Investigation, Software, Visualization, Writing – original draft. XJ: Software, Visualization, Writing – review & editing. HW: Writing – review & editing, Project administration, Resources. XH: Writing – review & editing, Supervision, Validation. HS: Writing – review & editing, Conceptualization, Formal analysis. CB: Resources, Visualization, Writing – original draft. LZ: Data curation, Funding acquisition, Methodology, Writing – review & editing. XW: Conceptualization, Methodology, Writing – original draft. RW: Formal analysis, Supervision, Writing – original draft.

## Funding

The author(s) declare financial support was received for the research, authorship, and/or publication of this article. This work was supported by the National Key R&D Program of China (grant no.: 2021YFE0101800); Natural Science Foundation of Jiangsu Province (grant no.: BK20221430); Jiangsu Agricultural Science and Technology Innovation Fund (grant no.: CX (22) 3003) and NZ MBIE Grant C03X1906.

## Conflict of interest

The authors declare that the research was conducted in the absence of any commercial or financial relationships that could be construed as a potential conflict of interest.

## Publisher’s note

All claims expressed in this article are solely those of the authors and do not necessarily represent those of their affiliated organizations, or those of the publisher, the editors and the reviewers. Any product that may be evaluated in this article, or claim that may be made by its manufacturer, is not guaranteed or endorsed by the publisher.

## Supplementary material

The Supplementary material for this article can be found online at: https://www.frontiersin.org/articles/10.3389/fmicb.2023.1266715/full#supplementary-material

## Footnotes

^1^ http://cab.spbu.ru/software/spades/

^2^ http://www.genomicepidemiology.org/

^3^ https://itol.embl.de/

^4^ https://pro-hunter.genomics.cn

^5^ https://rast.nmpdr.org/
